# The Effect of Epidural Analgesia on the Delivery Outcome of Induced Labour: A Retrospective Case Series

**DOI:** 10.1155/2016/5740534

**Published:** 2016-11-20

**Authors:** Angeliki Antonakou, Dimitrios Papoutsis

**Affiliations:** ^1^Department of Midwifery, Midwifery School, “Alexander” Technological Educational Institute of Thessaloniki, Thessaloniki, Greece; ^2^Department of Obstetrics and Gynaecology, Shrewsbury and Telford Hospitals NHS Trust, Shrewsbury, UK

## Abstract

*Objective*. To investigate whether the use of epidural analgesia during induced labour was a risk factor for instrumental vaginal delivery and caesarean section (CS) delivery.* Study Design*. This was a retrospective case series of primigravidae women being induced at term for all indications with a normal body mass index (BMI) at booking and under the age of 40 years.* Results*. We identified 1,046 women who fulfilled the inclusion criteria of which 31.2% had an epidural analgesia. Those with an epidural analgesia had significantly greater maternal age, higher BMI, greater percentage of oxytocin usage, and a longer first and second stage of labour. Women with an epidural analgesia had a higher instrumental delivery (37.9% versus 16.4%; *p* < 0.001) and CS delivery rate (26% versus 10.1%; *p* < 0.001). Multivariable analysis indicated that the use of an epidural was not a risk factor for a CS delivery but was a risk factor for an instrument-assisted delivery (adjusted OR = 3.63; 95% CI: 2.51–5.24; *p* < 0.001).* Conclusion*. Our study supports the literature evidence that the use of an epidural increases the instrumental delivery rates. It has also added that there is no effect on CS delivery and the observed increase is due to the presence of confounding factors.

## 1. Introduction

Epidural analgesia is a central nerve blockade technique which involves the injection of a local anaesthetic into the lower region of the spine, thus blocking the painful impulses that are generated from the nerves of the contracting uterus during labour. It is most commonly used for intrapartum pain management with approximately 20% of women in the United Kingdom [1] and 60% of women in the United States [2] utilising this technique as a form of pain relief. A recent Cochrane review in 2012 summarised the available evidence from other existing Cochrane systematic reviews on the efficacy and safety of nonpharmacological and pharmacological interventions to manage pain in labour [3]. The authors of this review reported that epidural analgesia is the most effective pain management method in comparison with other pharmacological and nonpharmacological methods [3]. However, even though the overall risk of a caesarean section (CS) delivery was not found to be increased, nevertheless epidural analgesia was found to be associated with an increased risk of assisted vaginal birth [3, 4].

The primary aim of our study was to investigate the effect of epidural analgesia on the delivery outcome in women with induced labour. In order to account for the significant confounding factors of parity [5], age [6], and body mass index (BMI) [7] on the success of induced labour, we restricted the inclusion criteria of our women to those who were primigravidae and under 40 years of age and had a normal BMI at booking.

## 2. Materials and Methods

This was a retrospective case series of women induced for all indications at term (gestational age ≥37 weeks) at the Maternity Unit of the Shrewsbury and Telford Hospital (SaTH) National Health Service (NHS) Trust, between January 2007 and December 2013. Primigravidae women with a normal body mass index (BMI) at booking (<25 kg/m^2^) and under the age of 40 years with singleton cephalic presentation deliveries were considered eligible for the study. Women induced for stillbirths and fetal congenital abnormalities and with multiple pregnancies were excluded. Data was collected from Medway® obstetric electronic database and maternal data, labour/delivery data, and neonatal data were all recorded.

Maternal data recorded involved age, body mass index at booking, smoking status, and self-reported ethnicity (White-European, Asian, Black, or other). Labour and delivery data included route of birth (normal vaginal delivery, instrumental vaginal delivery, or caesarean section delivery), indications for instrumental delivery and CS delivery, epidural analgesia use, and liquor appearance (normal, meconium stained). In our unit, epidural catheters are placed at the L2-L3, L3-L4, or L4-L5 interspace when women have a cervical dilatation of ≥3 cm. Finally, neonatal data recorded were fetal gender (male, female), birth weight, head circumference, Apgar scores (at 1 and 5 minutes), cord gases taken at delivery (arterial/venous pH), and admission to the neonatal unit (NNU).

Quantitative variables were expressed as mean values (SD, standard deviation) and qualitative variables were expressed as absolute and relative frequencies. For the comparison of proportions Fisher's exact tests were used, and Student's *t*-test was computed for the comparison of mean values. Multivariable logistic regression analyses in a stepwise method (*p* for entry 0.05, *p* for removal 0.10) were used in order to determine independent factors that were associated with the odds of an instrumental and caesarean section delivery. The variables that were entered in the primary analysis were time duration of first and second stage of labour, age of the mother, smoking, ethnicity, BMI, liquor appearance, use of epidural, fetal gender, birth weight, and head circumference at birth. Our study included 1,046 women and, with the current sample size, the study had >95% power to perform a logistic regression using an alpha of 0.05, large effect sizes, and two-tailed test. Statistical significance was set at *p* < 0.05 and analyses were conducted using SPSS statistical software (version 20.0).

Ethical approval for collection and analysis of data in our study was obtained by the Research and Development Department of the Shrewsbury and Telford Hospital NHS Trust.

## 3. Results

The total sample consisted of 1,046 eligible women with a mean maternal age at delivery of 25.9 years (SD = 5.7 years). 88.2% of women were of White ethnic background, 4.1% were Asian, and 1.1% were of Black ethnic background. The mean value of BMI was 22 kg/m^2^ (SD = 1.9 kg/m^2^) and 87.1% of the participants never smoked. During labour 31.2% of women had an epidural analgesia for pain relief and the instrumental delivery and overall caesarean section delivery rate were 23.1% and 15.1%, respectively. The mean birth weight was 3371 gr (SD = 559 gr) with 52.5% of the fetuses being male. Meconium stained liquor appearance was identified in 13.3% of the participants and 4% of all newborns were admitted to the neonatal unit (Tables 1 and 2).

The indications for an instrumental delivery (*n* = 242) were prolonged second stage (36.4%), cardiotocographic (CTG) abnormalities (36.4%), maternal exhaustion (15.2%), abnormal fetal blood sampling (FBS) (2.9%), fetal malposition (1.3%), and other indications such as eclampsia (0.8%), and there was a percentage of women with no indication recorded (7%). The indications for a CS delivery (*n* = 158) were failure to progress in labour (38.7%), CTG abnormalities (25.9%), failed instrumental delivery (12.7%), failed induction (10.7%), abnormal FBS (3.1%), and other indications (8.9%) such as chorioamnionitis and placental abruption.

Those with an epidural analgesia when compared to those without had a significantly greater maternal age, higher BMI, greater percentage of oxytocin usage, and a longer first and second stage of labour. Though all women had a normal BMI, the increasing BMI was associated with a greater use of oxytocin in labour (*p* = 0.01). The neonates of women with an epidural analgesia had a significantly greater birthweight and head circumference, lower Apgar scores at 1 minute but similar Apgar scores at 5 minutes, and higher values of arterial pH in their cord gases. Women with an epidural analgesia also had a significantly higher instrumental delivery (37.9% versus 16.4%; *p* < 0.001) and CS delivery rate (26% versus 10.1%; *p* < 0.001) (Tables 1 and 2).

Table 3 shows the results from multivariable stepwise logistic regression analysis with the dependent variable of presented route of birth (normal vaginal delivery versus instrumental delivery). The use of an epidural analgesia was independently associated with the odds of an instrumental vaginal delivery (OR = 3.63; 95% CI: 2.51–5.24, *p* < 0.001). Additionally, it was found that the increased mother's age at delivery, the increased second stage of labour, and decreasing gestational age were associated with greater odds for an instrumental delivery.

Table 4 presents the results from multivariable stepwise logistic regression analysis with the dependent variable of presented route of birth (vaginal delivery versus CS delivery). The use of an epidural analgesia was not found to be associated with the odds for a CS delivery. It was found that the increased birth weight and prolonged second stage were the two factors that increased the odds for CS delivery.

## 4. Discussion

We found that women with an epidural analgesia in comparison to those without had a significantly greater maternal age and a higher BMI. A survey conducted in 2010 showed that increasing maternal age was a significant factor associated with a woman's preference to have an epidural analgesia during labour [8]. A more recent, however, large-population based study in the United States demonstrated that distributions of age were similar between epidural users and nonusers [9]. On review of the literature, there are no studies directly reporting on the finding of increased rates of epidural analgesia in women with a higher BMI. Nevertheless, there are reports that the increased BMI due to the adipose tissue being hormonally active predisposes to a reduced response to the induction of labour process because of the altered metabolic status of these women [10, 11]. In our study we presume that women with a higher BMI may have also had a reduced response to induced labour, as we found that the increasing BMI was associated with a greater use of oxytocin in labour (*p* = 0.01) which could explain the higher rate of epidural usage due to a more painful labour.

Our study demonstrated that women with induced labour and an epidural analgesia as compared with those without had a significantly greater percentage of oxytocin usage and a longer first and second stage of labour. A recent Cochrane review in 2011 [4] reported that epidural analgesia was associated with an increased rate of oxytocin administration (RR = 1.19; 95% CI: 1.03–1.39). There is evidence that induced labour may be less efficient than spontaneous labour [12] and for this reason oxytocin administration may be necessary, thus rendering labour more painful and therefore requiring the use of pain relief. The Cochrane review in 2011 [4] also reported that epidural analgesia was associated with a longer second stage of labour (mean difference = 13.66 mins; 95% CI: 6.67–20.66) but showed no clear effect on the duration of first stage. On review of the literature there is conflicting evidence regarding the effect of epidural analgesia with reports of either prolonging [13] or shortening [14] the first stage of labour. In our cohort of women, both first and second stages of labour were prolonged in those women who had an epidural analgesia.

The neonates of women with epidural analgesia in our study when compared to those without had significantly lower Apgar scores at 1 minute but similar Apgar scores at 5 minutes. This is in line with the Cochrane review in 2011 [4] which reported that there were no significant differences in neonatal Apgar scores at 5 minutes in babies born to women with epidural analgesia. Our study has also shown that neonates from women with an epidural have significantly higher values of arterial pH in their cord gases. Higher cord pH values have also been reported in the past [15] and this finding could be explained by a recent immunohistochemical study [16] that demonstrated that pain-reducing anaesthesia seemed to reduce the oxidative stress in human term placenta.

We have found in our study that the use of an epidural analgesia after adjusting for multiple confounding factors was independently associated with the odds of an instrumental vaginal delivery (aOR = 3.63; 95% CI: 2.51–5.24). This is in line with the Cochrane review of 2011 [4] indicating an increased risk of assisted vaginal birth in women with an epidural during labour (RR = 1.42; 95% CI: 1.28–1.57). Previous studies however have shown that the rate of instrumental vaginal delivery depends on several other confounding factors such as the dose and concentration of the epidural solution used, the degree of analgesia during second stage, and obstetric factors [17, 18]. It has been reported that the motor block which is the chief complication of labour epidural analgesia might result in prolonged labour and therefore increase the rates of instrument-assisted delivery [19].

Women with an epidural analgesia in our study when compared to those without had a significantly higher CS delivery rate (26% versus 10.1%). Nevertheless, after adjusting for multiple confounding factors, there was no significant difference noted between epidural users and nonusers. This is in line with the Cochrane review of 2011 [4] indicating that there is no significant difference in the risk of CS delivery overall. Previous studies have contemplated that the degree of motor block achieved by an epidural analgesia may result in a prolonged labour and therefore increase the rates of a CS delivery [19]. Other studies [17, 20] however have demonstrated that epidural analgesia* per se* is unlikely to affect the chances of a normal delivery and there are many other factors that may contribute to a CS delivery such as the increased birthweight [17].

There are certain limitations to be considered about our study. First, data were retrospectively collected from an electronic database for the study period 2007–2013 where accuracy of data is dependent on the practitioner recording the information each time on the database. Second, our electronic database does not have a mandatory field for recording the epidural regimen that was used. There is literature evidence showing that different epidural analgesia formulas exhibit a different effect on the course of labour and the delivery outcome [19, 20]. The main strength of our study includes its large sample size with inclusion of women who were primigravidae and under 40 years of age and had a normal BMI at booking in order to account for the significant confounding factors of parity [5], age [6], and body mass index (BMI) [7] on the success of induced labour.

In conclusion we have found that women with an epidural in our cohort have a threefold increased risk of an instrumental delivery. Our study lends support to the literature reports that an epidural analgesia is a risk factor for an assisted vaginal birth. It has also added that there is no effect on the CS delivery rates and the observed increase is due to the presence of confounding factors.

## Figures and Tables

**Table 1:** Maternal demographics and labour/delivery characteristics.

	Total sample (*n* = 1,046)	Epidural, yes (*n* = 327)	Epidural, no (*n* = 719)	*p*
Mothers age at delivery (years), mean (SD)	25.9 (5.7)	26.4 (5.8)	25.6 (5.6)	0.039^†^
Ethnicity				
White ethnic background	920 (88.2%)	291 (89%)	629 (87.8%)	0.67^‡^
Asian ethnic background	43 (4.1%)	5 (1.6%)	34 (4.7%)	0.01^‡^
Black ethnic background	12 (1.1%)	9 (2.7%)	7 (0.9%)	0.05^‡^
Not stated	68 (6.6%)	22 (6.7%)	46 (6.6%)	0.89^‡^
BMI, mean (SD)	22.0 (1.9)	22.3 (1.9)	21.9 (1.9)	0.004^†^
Smoking				
No	893 (87.1%)	271 (85.5%)	622 (87.9%)	0.31^‡^
Yes	132 (12.9%)	46 (14.5%)	86 (12.1%)	
Gestation in days, mean (SD)	278 (13)	277 (13)	278 (13)	0.27^†^
Postdates pregnancy (≥41 weeks)				
No	664 (63.5%)	209 (64.5%)	718 (73%)	0.003^‡^
Yes	382 (36.5%)	115 (35.5%)	265 (26%)	
Route of birth				
Normal vaginal delivery	646 (61.8%)	118 (36.1%)	528 (73.5%)	0.0001^‡^
Instrumental vaginal delivery	242 (23.1%)	124 (37.9%)	118 (16.4%)	0.0001^‡^
Caesarean section delivery	158 (15.1%)	85 (26%)	73 (10.1%)	0.0001^‡^
Use of oxytocin				
No	790 (75.5%)	207 (63.3%)	583 (81.1%)	0.0001^‡^
Yes	256 (24.5%)	120 (36.7%)	136 (18.9%)	
First stage of labour (mins), mean (SD)	300 (211)	431 (239)	249 (174)	0.0001^†^
Second stage of labour (mins), mean (SD)	72 (62)	101 (69)	61 (56)	0.0001^†^
Liquor appearance				
Normal	902 (86.7%)	276 (84.9%)	616 (86.2%)	0.63^‡^
Meconium stained	138 (13.3%)	49 (15.1%)	99 (13.8%)	

^†^ Student's *t*-test; ^‡^ Fisher's exact test.

**Table 2:** Neonatal characteristics in the sample.

	Total sample (*n* = 1,046)	Epidural, yes (*n* = 327)	Epidural, no (*n* = 719)	*p*
Fetal gender				
Male	549 (52.5%)	178 (54.4%)	371 (51.6%)	0.42^‡^
Female	497 (47.5%)	149 (45.6%)	348 (48.4%)	
Birth weight (g), mean (SD)	3371 (559)	3483 (522)	3320 (568)	**<**0.001^†^
Birth weight (g)				
<4000	913 (87.3%)	268 (81.9%)	641 (89.3%)	0.002^‡^
≥4000	133 (12.7%)	59 (18.1%)	78 (10.7%)	
Head circumference at birth (cm), mean (SD)	34.7 (1.6)	35.0 (1.5)	34.6 (1.7)	**<**0.001^†^
Apgar score < 7 at 1 minute				
0–6	91 (8.7%)	37 (11.4%)	53 (7.4%)	0.002^‡^
7–10	949 (91.3%)	228 (88.6%)	662 (92.6%)	
Apgar score < 7 at 5 minutes				
0–6	14 (1.3%)	3 (0.9%)	11 (1.5%)	0.56^‡^
7–10	1025 (98.7%)	321 (99.1%)	704 (98.5%)	
Cord gases at delivery, arterial pH, mean (SD)	7.23 ± 0.07	7.24 ± 0.07	7.22 ± 0.08	0.04^†^
Cord gases at delivery, venous pH, mean (SD)	7.29 ± 0.06	7.29 ± 0.06	7.28 ± 0.07	0.07^†^
Admitted to NNU				
No	812 (96%)	242 (95.3%)	570 (96.3%)	0.56^‡^
Yes	34 (4%)	12 (4.7%)	22 (3.7%)	

^†^ Student's *t*-test; ^‡^ Fisher's exact test.

**Table 3:** Results from stepwise multivariable logistic regression analysis with the dependent variable presented route of birth (normal vaginal delivery (*N* = 646) versus instrumental delivery (*N* = 242)).

	*B* (SE)^*∗*^	OR (95% CI)^*∗∗*^	*p*
Time duration of second stage of labour (for 30 min increase)	0.32 (0.05)	1.38 (1.26–1.51)	<0.001
Mother's age at delivery (years)	0.05 (0.02)	1.05 (1.02–1.09)	0.001
Gestational age in days	0.02 (0.01)	0.98 (0.97–0.99)	<0.001
Epidural analgesia	1.29 (0.19)	3.63 (2.51–5.24)	<0.001

^*∗*^Regression coefficient (standard error). ^*∗∗*^Odds ratios (95% confidence interval).

**Table 4:** Results from stepwise multivariable logistic regression analysis with the dependent variable presented route of birth (vaginal delivery (*N* = 888) versus CS delivery (*N* = 158)).

	*B* (SE)^*∗*^	OR (95% CI)^*∗∗*^	*p*
Time duration of second stage of labour (for 30 min increase)	0.38 (0.07)	1.46 (1.27–1.68)	<0.001
Birth weight (for 100 g increase)	0.16 (0.04)	1.17 (1.08–1.27)	<0.001

^*∗*^Regression coefficient (standard error). ^*∗∗*^Odds ratios (95% confidence interval).
